# Low compression smart clothing for respiratory rate monitoring using a bending angle sensor based on double-layer capacitance

**DOI:** 10.1007/s13534-025-00456-w

**Published:** 2025-01-18

**Authors:** Tatsuya Kobayashi, Daisuke Goto, Yusuke Sakaue, Shima Okada, Naruhiro Shiozawa

**Affiliations:** 1 Graduate School of Sport and Health Science, Ritsumeikan University, Shiga, 525-8577 Japan; 2 Graduate School of Engineering Science, Osaka University, Toyonaka, 560-8531 Japan; 3 Department of Robotics, College of Science and Engineering, Ritsumeikan University, Shiga, 525-8577 Japan; 4 College of Sport and Health Science, Ritsumeikan University, Shiga, 525-5877 Japan

**Keywords:** Smart clothing, Respiratory rate, Low compression, Bending angle sensor

## Abstract

In chronic respiratory diseases, continuous self-monitoring of vital signs such as respiratory rate aids in the early detection of exacerbations. In recent years, the development of smart clothing, such as garments equipped with sensors to measure respiratory rate, has been a focus of research. However, the usability and adoption of smart clothing are often compromised owing to the discomfort caused by compression pressure during wear. This study developed smart clothing designed to measure respiratory rate using a low compression pressure. This was achieved by integrating a bending angle sensor, based on double-layer capacitance, into the rib cage and abdomen areas. The accuracy of the respiratory rate measurement was evaluated in 20 healthy male subjects without respiratory diseases. Breathing was measured while the subjects wore the smart clothing and performed breathing exercises in sitting, supine, and lateral postures, following a metronome set between 12 and 30 bpm. To assess accuracy, the respiratory rate measured by the smart clothing was compared with that measured by a spirometer. The recorded compression pressure was 0.77 ± 0.21 kPa, with no subjects reporting discomfort. Correlation coefficients for respiratory rate in the different postures ranged within 0.97–0.99. The mean difference between the smart clothing and spirometer measurements was less than 0.1 bpm. The low mean difference indicated that the proposed low compression pressure wearable respiration sensor, employing a bending angle sensor based on double-layer capacitance, could measure respiratory rate accurately without causing discomfort and within an acceptable error range.

## Introduction

Chronic respiratory diseases impact over 570 million individuals globally, as reported by the World Health Organization. Asthma and chronic obstructive pulmonary disease (COPD) are among the most prevalent of these conditions [1]. Symptoms common to many chronic respiratory diseases, such as asthma and COPD, include coughing, phlegm production, and reduced airflow due to bronchial constriction. In addition, inadequately controlled symptoms have been linked to increased healthcare costs [2] and diminished quality of life for patients [3]. Self-monitoring by patients has been shown to effectively manage symptoms of chronic respiratory diseases, potentially reducing severity through medication adjustments and behavioral changes [4, 5]. Continuous monitoring of vital signs is beneficial for the early detection of symptom exacerbation [6], with daily respiratory rate being a crucial marker alongside blood pressure and body temperature [7–9]. However, measuring respiratory rate traditionally involves counting breaths per minute manually, a task that can be challenging for individuals.

Respiratory rate measurement methods are generally categorized into two types: wearable devices worn by the individual, and environmental systems positioned within the individual’s surroundings [10]. Environmental methods include placing a mat on a bed [11] or utilizing cameras or radar systems [12]; however, these are limited by their dependency on specific settings such as a room or bed. In terms of wearable technology, methods have been devised to measure airflow changes caused by breathing around the mouth and nose to measure breath sounds, heartbeat and pulse wave changes, and chest movements [13]. Methods for measuring airflow changes require sensors to be attached around the mouth and nose. Mask-type sensors have been studied [14], but wearing a mask for long periods of time can be suffocating and uncomfortable. The methods that measure breath sounds have low signal-to-noise (S/N) ratio due to environmental noise and low breath sounds. In addition, the inclusion of rubbing sound due to misalignment of the device can be problematic. The methods that measure changes in heartbeat and pulse wave have poor tracking performance for irregular breathing, limiting their use to only specific situations. Extensive research has been conducted on smart clothing with integrated sensors to detect movements of the rib cage and abdomen [15]. Furthermore, there are various types of sensors [16]. In the method of measuring impedance, electrodes are attached to the chest cavity to measure impedance changes owing to respiration [17, 18]; however, this requires the electrodes to be in constant and stable contact with the chest cavity. Accelerometers have been used to measure movements of the thorax and abdomen [19–21]; however, they must be wrapped with a band or attached directly to the living body. Traditional methods employ capacitance sensors to detect pressure variations [22–26], piezoresistive sensors for measuring stretch [27, 28], or fiber optic sensors [29, 30], necessitating close-fitting sensors which can cause discomfort. A compression pressure exceeding 5.88 kPa, near venous pressure, is perceived as uncomfortable by most individuals, and deep breathing in smart garments using capacitance sensors typically produces pressures between 3 and 7 kPa [22], surpassing this discomfort threshold [31]. Such high pressures might discourage people from wearing smart garments. Therefore, an alternative method to measure the respiratory rate without involving uncomfortable pressure levels is needed.

This study introduces the use of a bending angle sensor based on double-layer capacitance in smart clothing for respiratory rate measurement with low compression levels. The performance of this bending angle sensor was evaluated through the measurement of knee joint angles during cycling [32]. The feature of this sensor is that, in principle, the total bending angle of the sensor becomes the change in capacitance, and as the sensor measures positive and negative bending angles in a double layer, the bending angle can be accurately measured even if the sensor is bent or wrinkled during use. Therefore, the bending angle is measured according to changes in body shape even if the smart garment is wrinkled with low wearing pressure. Given its high precision and resistance to distortions from sensor wrinkles, the integration of the bending angle sensor into smart clothing could enable accurate monitoring of body shape changes due to respiration without the discomfort associated with high compression levels. The key contribution of this study is the development of smart clothing that enables continuous self-monitoring of vital signs without uncomfortable pressure, which can be useful for early detection of symptom exacerbations.

## Materials and methods

### Bending angle sensor

**Fig. 1:**
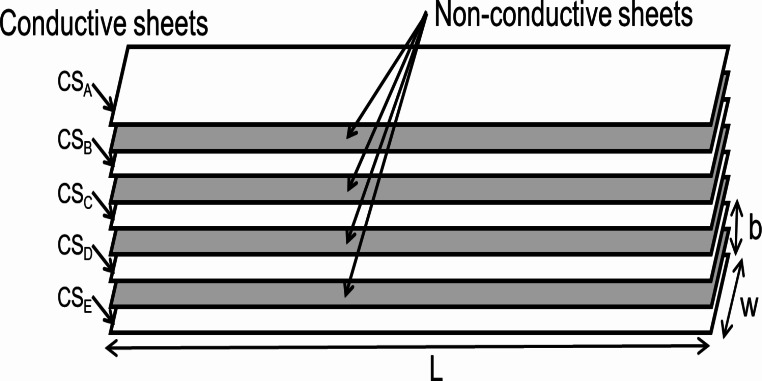
Bending angle sensor based on double-layer capacitance. Structure of the bending angle sensor, featuring five conductive layers separated by nonconductive layers. The dimensions of the sensor are 600 mm × 20 mm (length (L) × width (W))

**Fig. 2:**
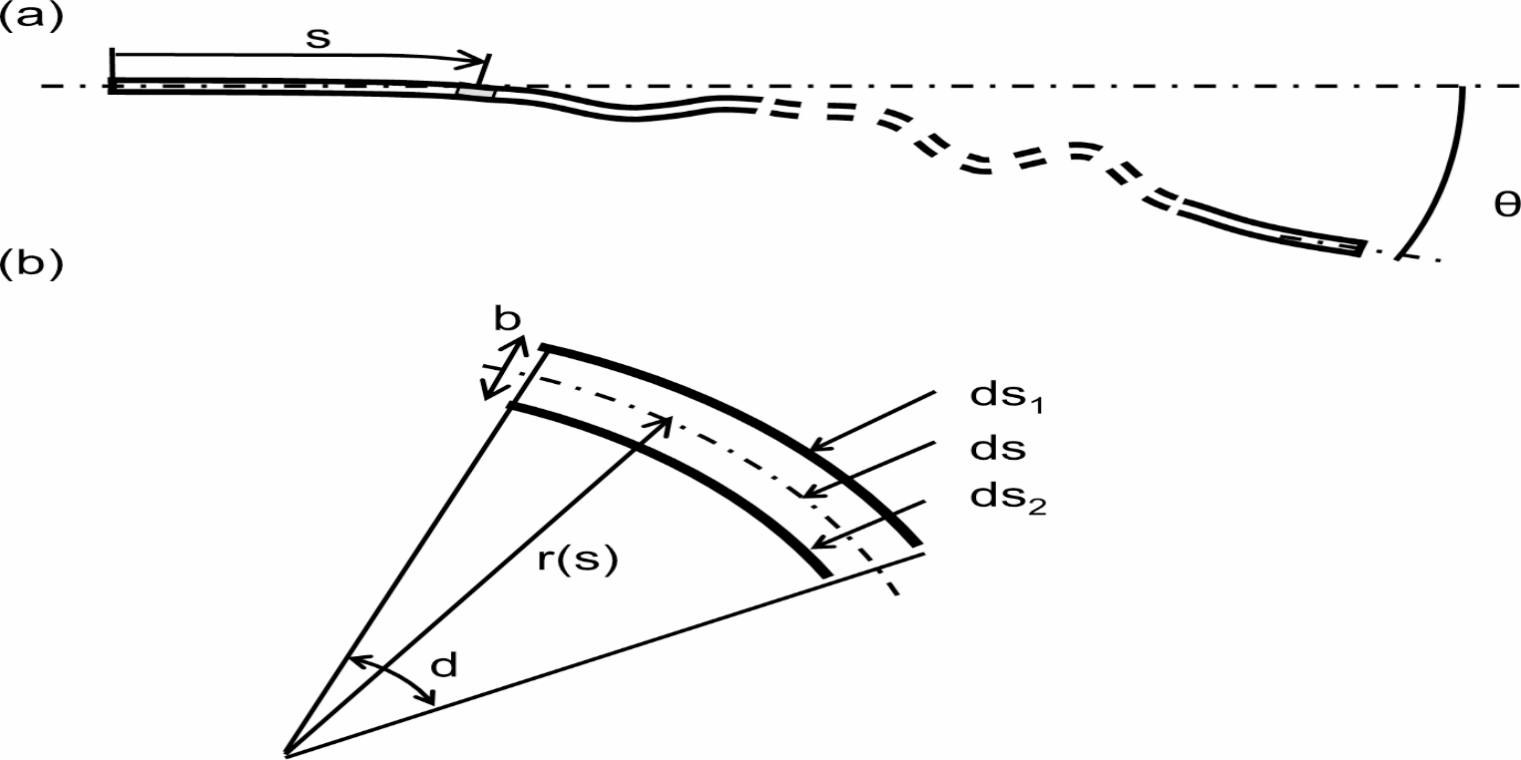
Illustration of bending angle and capacitance variation. (**a**) The entirety of conductive sheets and (**b**) Enlarged view of the micro-interval section

Figure 1 shows the layered structure of the bending angle sensor based on the double-layer capacitance technology. The sensor design is based on the one used in a previous study [32]. It comprises five layers, alternating between conductive (CS_A_ to CS_E_) and nonconductive materials. The length of the sensor is more than 600 mm, which surpasses the typical half circumference of the rib cage and abdomen, ensuring comprehensive coverage. Its width is maintained at 20 mm to facilitate easy integration with smart clothing. The capacitance between successive conductive sheets is calculated using the following equation:

1 $$\:\begin{array}{c}C={\epsilon\:}_{0}{\epsilon\:}_{r}\frac{wl}{b},\end{array}$$

where *C* is the capacitance, $\:{\epsilon\:}_{0}$ and $\:{\epsilon\:}_{r}\:$are the permittivity of vacuum and a nonconductive sheet, respectively, *l* is the length, *w* is the width, and *b* is distance between successive conductive sheets. Consider a micro-interval section ds in which the angle between both ends of the pair of conductive sheets is *θ* and the deformation is discretionary (Fig. 2a). When the radius of curvature in the micro-interval is *r(s)* from one end to a length s, each conductive sheet is distorted by a length change rate of $\:{\epsilon\:}_{1}$ and $\:{\epsilon\:}_{2}$ in the micro-interval *ds*. The two distortions, assuming that they are on a circular arc as shown in Fig. 2b, can be computed using the following equations:

2 $$\:\begin{array}{c}{\epsilon\:}_{1}=\frac{{ds}_{1}-ds}{ds}=\frac{b}{2r\left(s\right)},\:\:\end{array}$$

3 $$\:\begin{array}{c}{\epsilon\:}_{2}=\frac{{ds}_{2}-ds}{ds}=-\frac{b}{2r\left(s\right)},\:\end{array}$$

where $\:{\epsilon\:}_{1}$ and $\:{\epsilon\:}_{2}$ are the length change rate. The expansion and contraction rates $\:{{\Delta\:}l}_{1}$ and $\:{{\Delta\:}l}_{2}$ for the entire measurement section can be obtained by integrating $\:{\epsilon\:}_{1}$ and $\:{\epsilon\:}_{2}$, respectively.

4 $$ \begin{aligned} {\Delta }l_{1} & = \mathop \int \limits_{0}^{l} \varepsilon _{1} ds = \mathop \int \limits_{0}^{\theta } \frac{b}{{2r\left( s \right)}}r\left( s \right)d\theta \\ & = \frac{b}{2}\theta, \\ \end{aligned} $$

5 $$ \begin{aligned} {{\Delta }}l_{2} = & \mathop \int \limits_{0}^{l} \varepsilon _{2} ds = \mathop \int \limits_{0}^{\theta } - \frac{b}{{2r\left( s \right)}}r\left( s \right)d\theta \\ = & - \frac{b}{2}\theta, \\ \end{aligned} $$

Therefore, regardless of the shape of the deformation of the conductive sheet, the amount of expansion and contraction is proportional to the angle θ between the two ends. As shown in Eq. (1), since the capacitance is proportional to the length of the conductive sheet, the capacitance is also proportional to the angle *θ* between the two ends, regardless of the shape of the deformation of the conductive sheet.

6 $$ \begin{aligned} \Delta C &= \varepsilon _{0} \varepsilon _{r} \frac{{w\left( {l + {{\Delta }}l_{1} } \right)}}{d} - \varepsilon _{0} \varepsilon _{r} \frac{{w\left( {l + {{\Delta }}l_{2} } \right)}}{d} \\ &= 2\varepsilon _{0} \varepsilon _{r} w\theta, \\ \end{aligned} $$

Therefore, only the bending angle θ can be measured accurately even if the conductive sheet is wrinkled. In this study, the conductive sheet has a five-layer structure, and the capacitance between each layer is C_A−B_, C_B−C_, C_C−D_, and C_D−E_. The uppermost surface CS_A_ and the lowermost surface CS_E_ are connected to the ground (GND) of the measurement circuit to isolate the capacitance of the sensor from the influence of the human body and environmental potentials. Therefore, the capacitance difference (ΔC) between the parallel capacitances of C_A−B_ and C_B−C_ (C_U_) and C_C−D_ and C_D−E_ (C_L_) is measured. Even if the bending angle sensor is compressed or stretched, ΔC is not affected because C_U_ and C_L_ change in identical manner. The bending angle sensor of the double-layer capacitance method can accurately measure only the bending angle θ, independent of the shape change due to length, pressure, or wrinkle.

### Smart clothing for respiratory rate monitoring

**Fig. 3:**
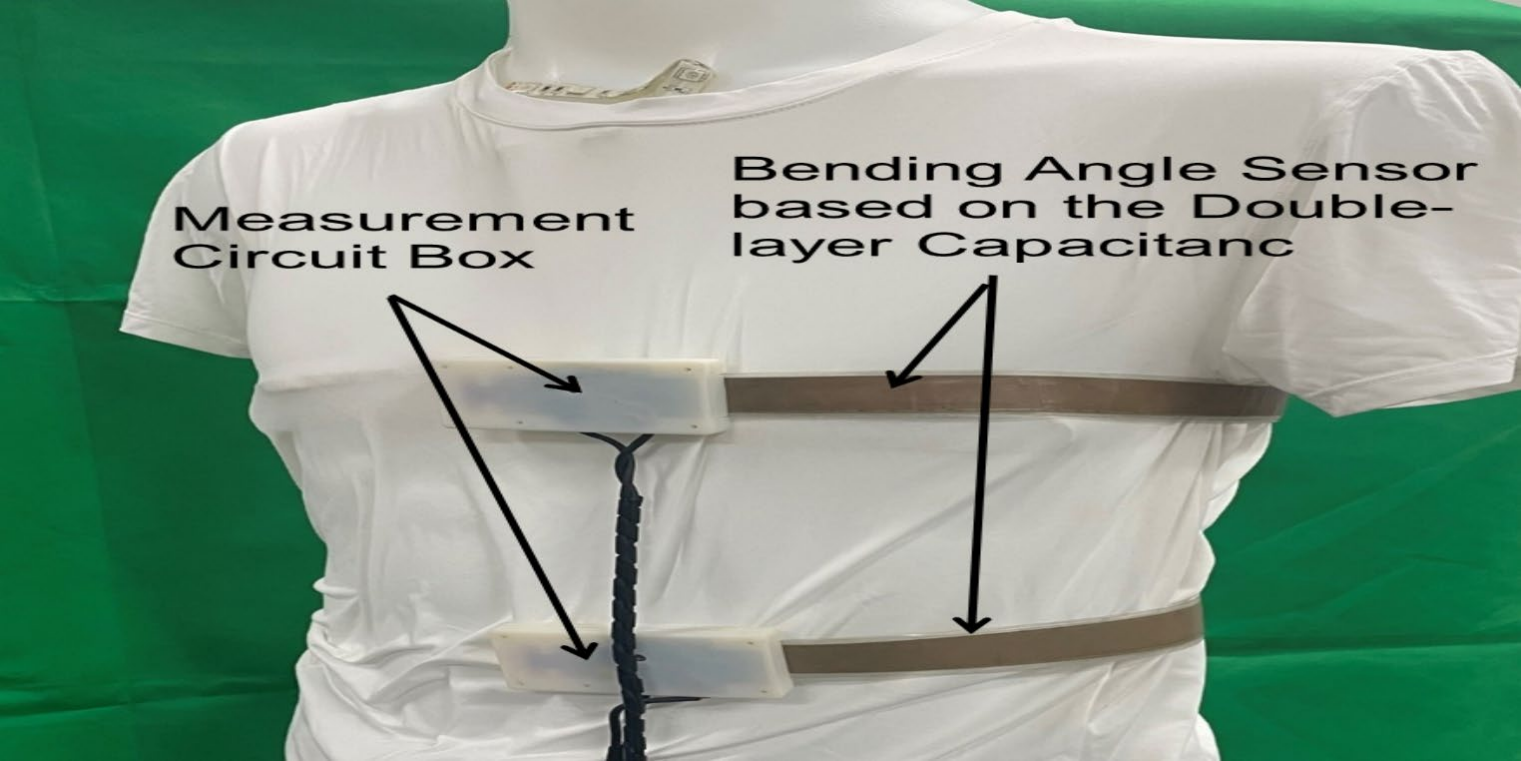
Illustration of smart clothing equipped with a bending angle sensor based on double-layer capacitance

Figure 3 shows a low compression smart clothing integrated with the proposed bending angle sensor. In a previous study, changes in the body shape in the rib cage and abdomen regions during resting breathing were found to vary with posture [33]. For this reason, in the present study, the bending angle sensors were strategically positioned at the height of the rib cage and abdomen, extending from the center of the front side to the back, to optimize the detection of respiratory movements. The measurement circuit box was centrally located on the front side of the garment. The bending angle sensor and measurement circuit box were attached to the underwear using double-sided tape.

**Fig. 4:**
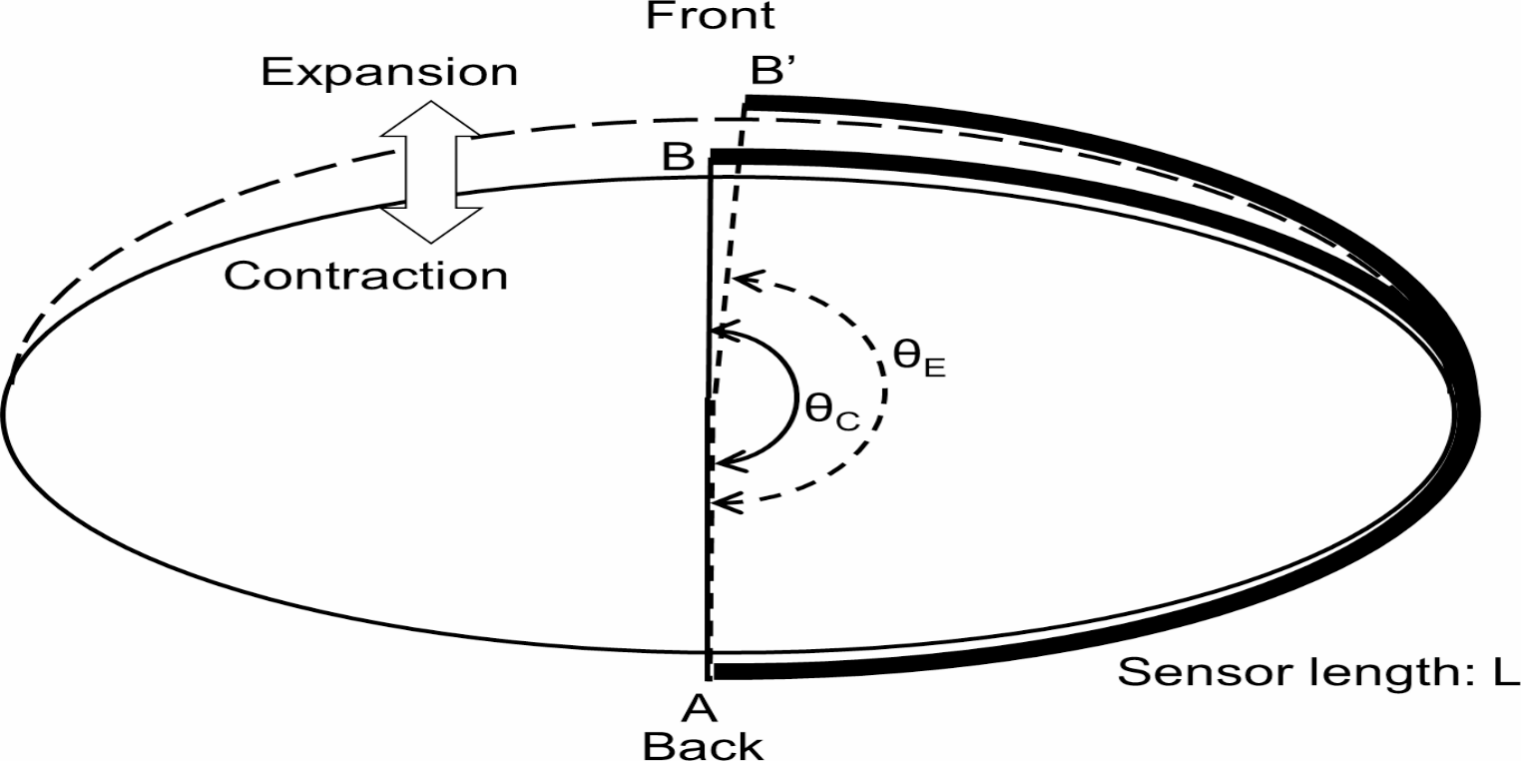
Schematic illustrating the bending angle sensor based on double-layer capacitance responding to changes in body shape owing to breathing. The solid line represents the body shape at exhalation, and the dashed line represents the expanded body shape at inhalation. The bending angle of the sensor changes accordingly from θ_C_ to θ_E_

Figure 4 shows a schematic of the alterations in body shape during respiration, as viewed in a horizontal cross-section. The bold line represents the sensor, whereas the solid and dashed lines represent the body contours during inhalation and exhalation, respectively. Notably, the double-layer capacitive-type bending angle sensor used in this research can measure the bending angle θ at both ends without being affected by the shape change in the middle of the sensor; therefore, there is no need to consider the shape in the middle of the sensor despite the proposed low-pressure smart garment causing wrinkles in the bending angle sensor. Furthermore, the sensor length (L) remains constant despite variations in body shape caused by breathing because the tension in the sensor exceeds the wearing pressure. This results in a shift of the position of the sensor from A-B during exhalation to A-B’ during inhalation. Accordingly, the angle of the sensor changes from $\:{\theta\:}_{C}$ to $\:{\theta\:}_{E}$ owing to respiratory movements. The corresponding change in capacitance induced by breathing, denoted as $\:\varDelta\:{C}_{resp}$, is quantified using Eq. (7).

7 $$\:\begin{array}{c}\varDelta\:{C}_{resp}=2{\epsilon\:}_{0}{\epsilon\:}_{r}w\left({\theta\:}_{C}-{\theta\:}_{E}\right)\end{array}$$

### Respiratory rate monitoring system

**Fig. 5:**
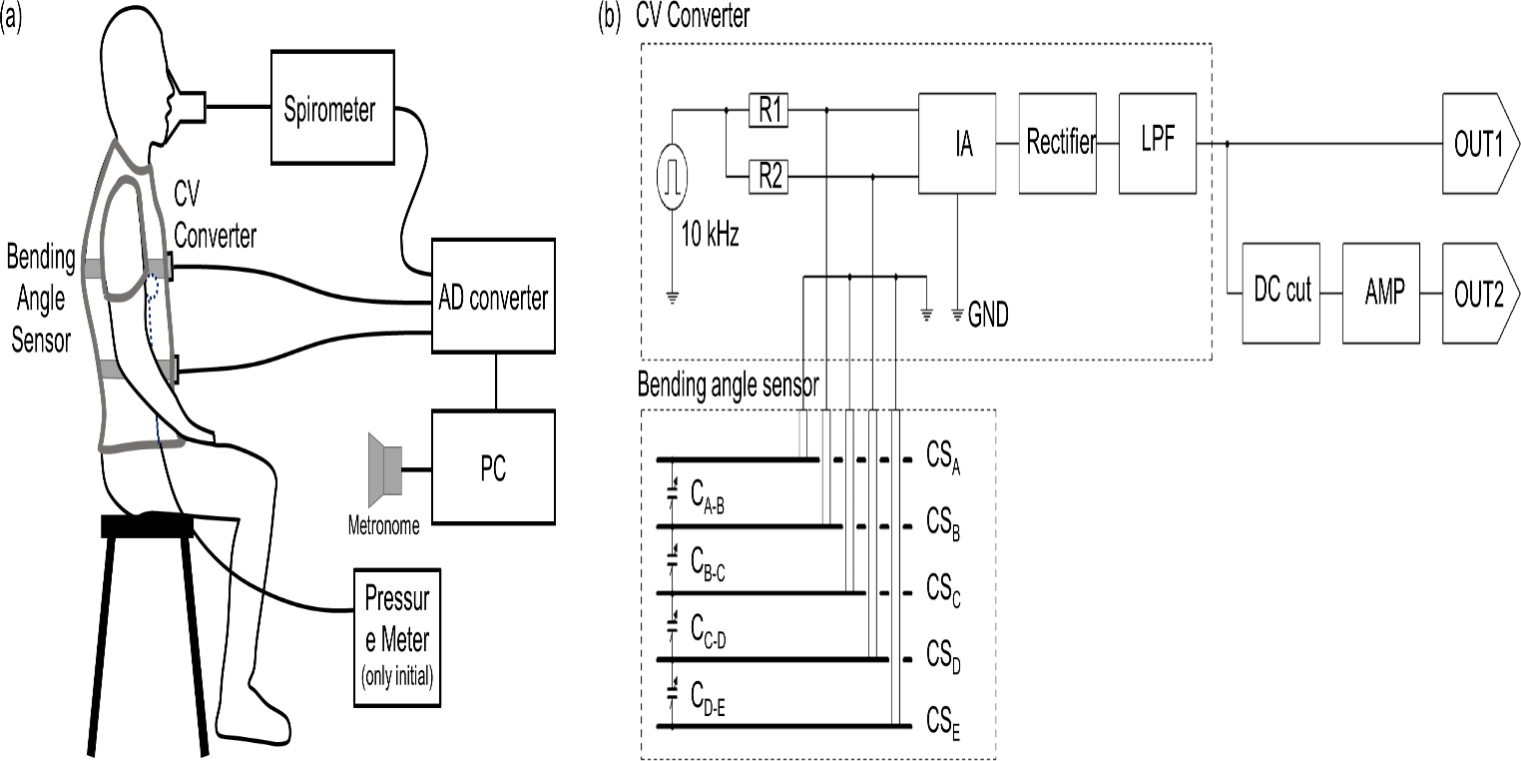
Illustration of experimental setup and measurement block diagram. (**a**) An example of measurement in the sitting posture. Pressure meter and air bag are used only initially and removed before the respiratory rate measurement. (**b**) Measurement block diagram for respiratory rate monitoring, featuring the bending angle sensor based on double-layer capacitance coupled with a CV converter

Figure 5 shows the experimental setup and measurement block diagram. The sensor output signals from the smart clothing positioned at the rib cage and abdomen, along with the ventilation volumes from the spirometer, were simultaneously recorded on a PC through an analog-to-digital (AD) converter (NI USB-6128, National Instruments) at a sampling frequency of 100 Hz. Simultaneously, ventilation volume was measured using an electronic spirometry system equipped with a gas analyzer (AE-310 S, Minato Medical Science, Osaka, Japan). In addition, a metronome controlled via a PC indicated the respiration timing. The compressive pressure exerted by the sensors on the thorax was measured using the airbag method [34]. This setup was used to monitor the respiratory rate in different postures.

Figure 5b shows the respiratory rate monitoring system that incorporates the bending angle sensor. In this system, the capacitance of the sensor is converted into voltage via a capacitance-to-voltage (CV) converter. The conductive sheets CS_A_, CS_C_, and CS_E_ of the sensor are connected to the ground (GND) of the CV converter. The CV converter applies a 10 kHz pulse wave through resistors R1 and R2 to the sensor. The charge and discharge voltages are fed into an instrumentation amplifier (IA) and subsequently processed through a half-wave rectifier circuit and a low-pass filter (LPF), which has a cutoff frequency above 10 Hz. The resulting output signal (OUT1) translates the differences in parallel capacitance between C_A−B_+C_B−C_ and C_C−D_+C_D−E_ into voltage, capable of detecting both positive and negative bending angles of the sensor. The output signal for respiratory detection (OUT2) undergoes further signal processing, including DC cut and amplification to enhance its reliability and accuracy.

### Signal processing

Bending angle sensors also pick nonrespiratory signals, such as body motion. Therefore, at the onset of the signal processing, we identified periods affected by body motion in the sensor output to prevent false detections. High-pass filter (HPF) processing with a cutoff frequency of 3 Hz, which can attenuate respiratory signal components of 0.2–0.5 Hz to 1/10 or less, was applied to the ADC1 signals obtained from the rib cage and abdomen sensors to detect noise related to body movements. Periods influenced by body motion were designated as 1 s before and after the detection of an amplitude exceeding a predefined threshold value post-HPF processing. Subsequently, band-pass Filter (BPF) processing with a frequency range of 0.2–0.5 Hz, which is the respiratory frequency measurement range, was applied to the ADC2 signals obtained from the rib cage and abdomen sensors. During this stage, periods influenced by body motion were ignored by treating them as no-signal periods. In this case, the period affected by body motion was ignored as a no-signal period. Because the chest and abdominal movements due to breathing in normal subjects are synchronized [35], to stabilize the waveform, we averaged the signals from the rib cage and abdomen to reduce the noise occurring specifically in the thorax or abdomen. However, because the amplitude of each sensor signal was different, we normalized the signals obtained from both the thorax and abdomen after BPF processing and then generated the ensemble waveform. The expiratory and inspiratory phases were identified within the ensemble waveform using the zero-crossing method [10]. This study aimed to measure the respiratory rate at rest and it was calculated using a moving average of the detected respiratory intervals. A three-point moving average was used wherein the difference of respiratory rate within the moving average points was within ±1 bpm owing to the use of the metronome to cue the respiratory rate acceleration by 1/3 bpm with each breath. For comparison, the respiratory rate was also estimated from the respiratory waveform obtained using the spirometer. The amplitude of the ensemble waveform at each breath was also computed.

### Subjects and experimental procedure

Twenty healthy male subjects without any respiratory diseases, including asthma and COPD, participated in this study to evaluate the performance of respiratory rate measurement using smart clothing. The study received ethical clearance from the Ethical Review Committee of Ritsumeikan University (Approval number BKC-LSMH-2022-072), and all participants provided informed consent prior to participation. Details regarding the subjects are summarized in Table 1.

**Table 1:** Subject information

Participant demographics	Mean ± SD
Age (years)	24.0 ± 1.9
Height (cm)	169.2 ± 6.4
Weight (kg)	65.3 ± 15.4
BMI (kg/m^2^)	22.7 ± 4.4
Circumference of rib cage (cm)	89.3 ± 10.2
Circumference of abdomen (cm)	82.2 ± 9.9

SD: Standard deviation

Initially, all subjects wore the smart clothing, and the compression pressure exerted by the sensor at the rib cage was assessed using the air bag method [34]. The compression pressure under the smart clothing at the rib cage sensor was measured using a Φ15-mm air bag to be in the range 0.05–2.00 kPa. Subjects also verbally confirmed whether they experienced any discomfort due to the compression pressure. Subsequently, participants were instructed to breathe for approximately 3 min in sitting, supine, and lateral postures, synchronized with a metronome controlled via a PC that gradually increased the respiratory rate from 12 bpm to 30 bpm. The lateral position was defined as the position where the bending angle sensor was placed between the bed and body. Because the sensor was under pressure, this configuration was selected as a more severe condition than that in the opposite direction.

The measurement accuracy was assessed through a Bland–Altman analysis, which compared the respiratory rates derived from the low compression smart clothing and the spirometer to find out the similarity. Furthermore, the mean absolute error (MAE) and mean absolute percentage error (MAPE) were used as additional error measures to show the normalized errors. According to a previous study [36], the 95% limits of agreement for respiratory rate measurements by the same, different, and simultaneous observers were −4.86 to 4.94 bpm, −5.7 to 5.7 bpm, and −4.2 to 4.4 bpm, respectively. In this study, we calculated the percentage of respiratory rates that fell within a 4 bpm error margin. Additionally, to explore the relationship between compression pressure and measurement error, we compared the pressure against the mean limit of agreement (LOA) across all postures for each subject. The Pearson correlation coefficient was utilized to determine statistical significance, with a threshold of *p* < 0.05 for significant results.

## Results

### Compression

The compression pressure exerted by the sensor at the rib cage was measured using the air bag method. The mean and standard deviation (SD) of the compression pressure recorded across all subjects was 0.77 ± 0.21 kPa. The maximum compression pressure was 1.16 kPa, significantly below the discomfort threshold of 5.88 kPa. Consequently, none of the subjects reported discomfort owing to the compression pressure.

### Waveforms

**Fig. 6:**
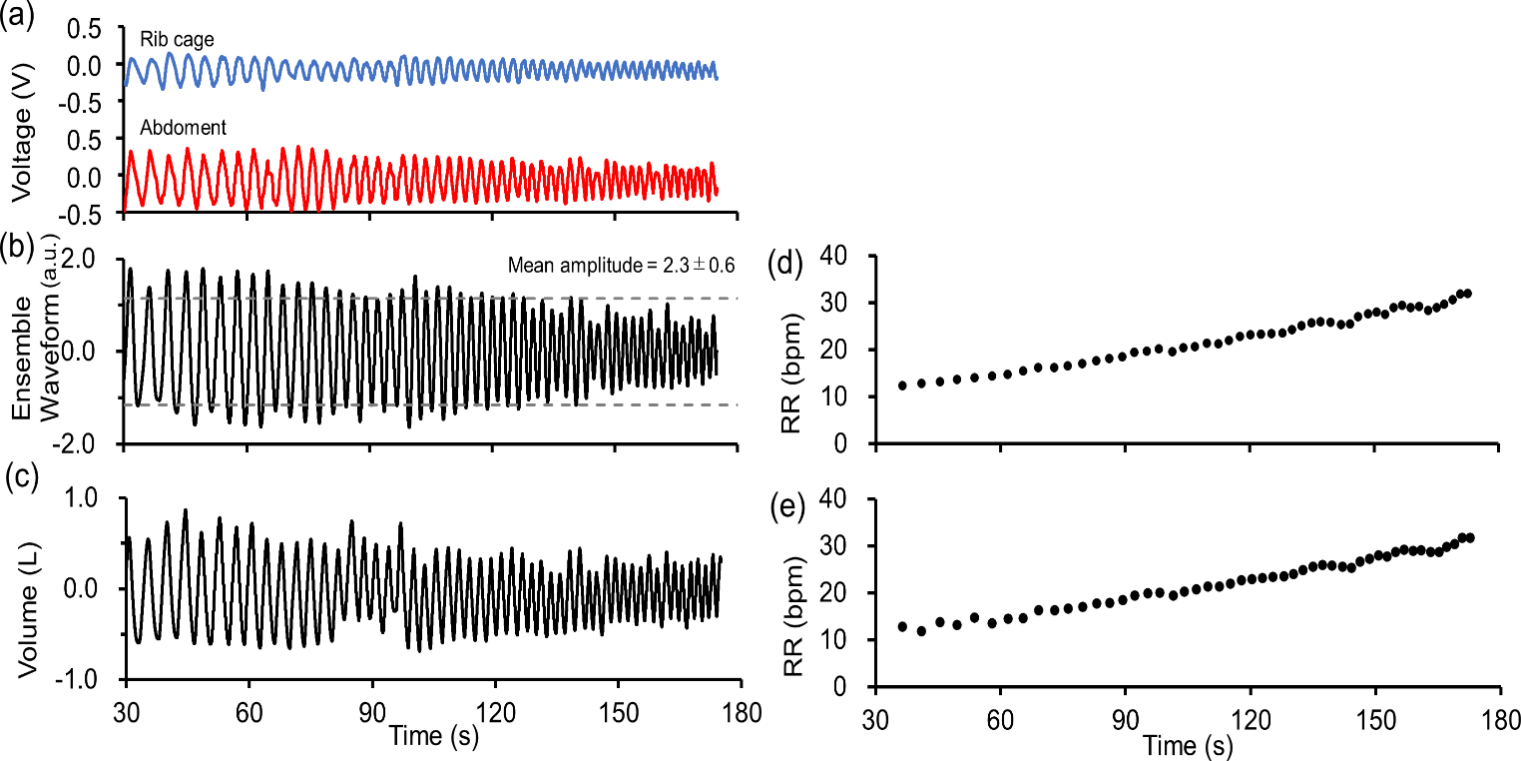
Measured respiration signal and respiratory rate. (**a**) Sensor output for rib cage (upper) and abdomen (lower). (**b**) Ensemble waveform of rib cage and abdomen. The amplitude of breathing was 2.3 ± 0.6 (mean ± SD). (**c**) Ventilation reading using a spirometer. (**d**) Respiratory rate calculated from sensor output. (**e**) Respiratory rate derived from the ventilation rate

Respiratory rate measurements were conducted using the bending angle senser and spirometer for approximately 3 min in sitting, supine, and lateral postures. The respiratory rate varied as 12–30 bpm. Figure 6 shows the measured waveforms and respiratory rates. Figure 6a shows the outputs from the sensor at the rib cage (depicted in blue) and abdomen (depicted in red). Figure 6b shows the normalized ensemble waveform of the rib cage and abdomen after BPF processing. Figure 6c shows the ventilation volume measurements from the spirometer. Figure 6d shows the respiratory rates calculated from the output waveforms of both sensors. Figure 6e shows the respiratory rates calculated based on the spirometer data.

During the measurements, subjects breathed in sync with a metronome, which led to a gradual increase in respiratory rate from 12 to 30 bpm. Owing to the forced changes in the respiratory rate during resting breathing, as the respiratory rate increased, the ventilation volume for each breath decreased. This pattern was similarly reflected in the sensor outputs from both the rib cage and abdomen, where the signal amplitude decreased as the respiratory rate accelerated. In Fig. 6b, the amplitude of the ensemble waveform during each breath was 2.3 ± 0.6 a.u. (mean ± SD). The mean amplitude for all subjects was 2.2 a.u. and the maximum– minimum range was 4.5–0.3 a.u.

### Performance evaluation

**Fig. 7:**
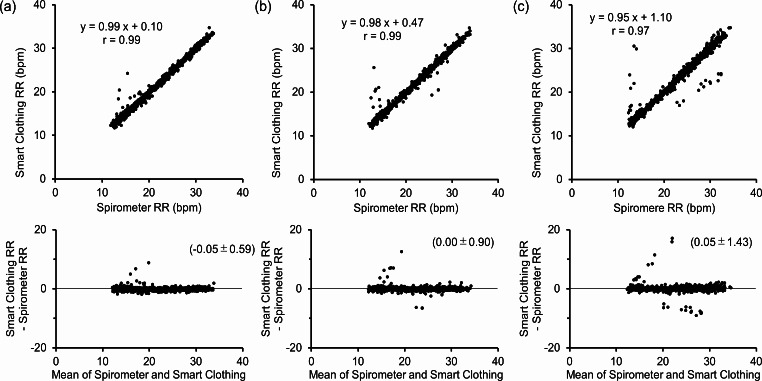
Comparison of respiratory rate measurements between smart clothing and a spirometer in sitting, supine, and lateral postures. (**a**) Comparison in the sitting position and Bland–Altman plot for the sitting position. (**b**) Comparison in the supine position and Bland–Altman plot for the supine position. (**c**) Comparison in the lateral position and Bland–Altman plot for the lateral position

The respiratory rate measurements were analyzed using the Bland–Altman technique. Figure 7 shows scatter plots and Bland–Altman plots comparing the respiratory rates measured using smart clothing and spirometer for three different postures: sitting (Fig. 7a), supine (Fig. 7b), and lateral (Fig. 7c). The correlation coefficients (r) for the sitting, supine, and lateral postures were statistically significant (0.99, 0.99, and 0.97, *p* < 0.001, respectively). The Bland–Altman analysis revealed errors of -0.05 ± 0.59, 0.00 ± 0.90, and 0.05 ± 1.43 for the sitting, supine, and lateral postures, respectively. The MAE and MAPE were 0.33 and 1.7%, 0.40 and 2.0%, and 0.55 and 2.7%, respectively. The percentage of errors within 4 bpm of the respiratory rate were 99.7, 98.9, and 97.8% for the sitting, supine, and lateral postures, respectively. Six out of 55 data points presented an error (LOA) of 4 bpm or more in all postures.

## Discussion

In this study, we proposed a method for measuring respiratory rate at low compression using smart clothing equipped with a bending angle sensor based on double-layer capacitance. We verified the accuracy of measuring respiratory rates to be within 12–30 bpm in the resting, supine, and lateral postures. The correlation coefficients between the measurements from the smart clothing and the spirometer in each posture were above 0.97 and these correlations were statistically significant for all postures. The maximum LOA in each posture was 2.8. Furthermore, the maximum SD for each posture and maximum MAE were 1.43 and 0.55, respectively, which were equivalent to or worse than the results reported in previous studies using a capacitive sensor or commercially smart textile (Table 2). However, the average difference between the measurements taken with smart clothing and the spirometer was within 0.1 bpm, which was consistent with the results of previous studies and equivalent to that of commercially available smart clothing [37]. Furthermore, the LOA for the concurrent observer group was in the range of -4.2–4.4 bpm [36]; thus, the results of this study were lower than those of previous studies. Therefore, the proposed method has better performance than the direct counting method in measuring the resting respiratory rate, and the proposed system can be practically applied.

**Table 2:** Comparison of the respiratory rate results

Study	Methods	Posture	Mean ± SD	MAE	Compression Pressure (kPa)
This study	Bending angle	Sitting	-0.05 ± 0.59	0.33	0.77 ± 0.21
		Supine	0.00 ± 0.90	0.40	
		Lateral	0.05 ± 1.43	0.55	
Min et al. [25]	Force or pressure	-	-0.0015 ± 0.2568	-	-
Naranjo et al. [26]	Body capacitance	-	-0.14 ± 0.28	-	-
Elliot et al. [27]	Commercially	-	-	1.6	-
Lim et al. [36]	Simultaneous observer	-	0.1 ± 2.2	-	-

SD: Standard deviation, MAE: Mean absolute Error

In a previous study that measured breathing using smart clothing equipped with capacitive sensing technology [22], high pressure had to be applied, which caused discomfort to the body. In contrast, the respiration measurement method using the proposed smart clothing in this study could measure the respiration rate effectively even under low wearing pressure. In the validation experiment, subjects with a BMI range of 18.4–35.3, chest circumferences within 78.5–114.5 cm, and abdominal circumferences within 67.5–107.0 cm were successfully measured for respiratory rate when the wearing pressure was between 0.45 and 1.16 kPa. No subject reported discomfort from compression pressure. Although compression pressures exceeding venous pressure can cause discomfort [31], our smart clothing likely avoided discomfort by maintaining pressure below this threshold. Therefore, the respiratory rate measurement method proposed in this study offers minimal user burden, flexible measurement conditions, and is unaffected by the user’s body shape, potentially rendering it suitable for prolonged monitoring.

**Fig. 8:**
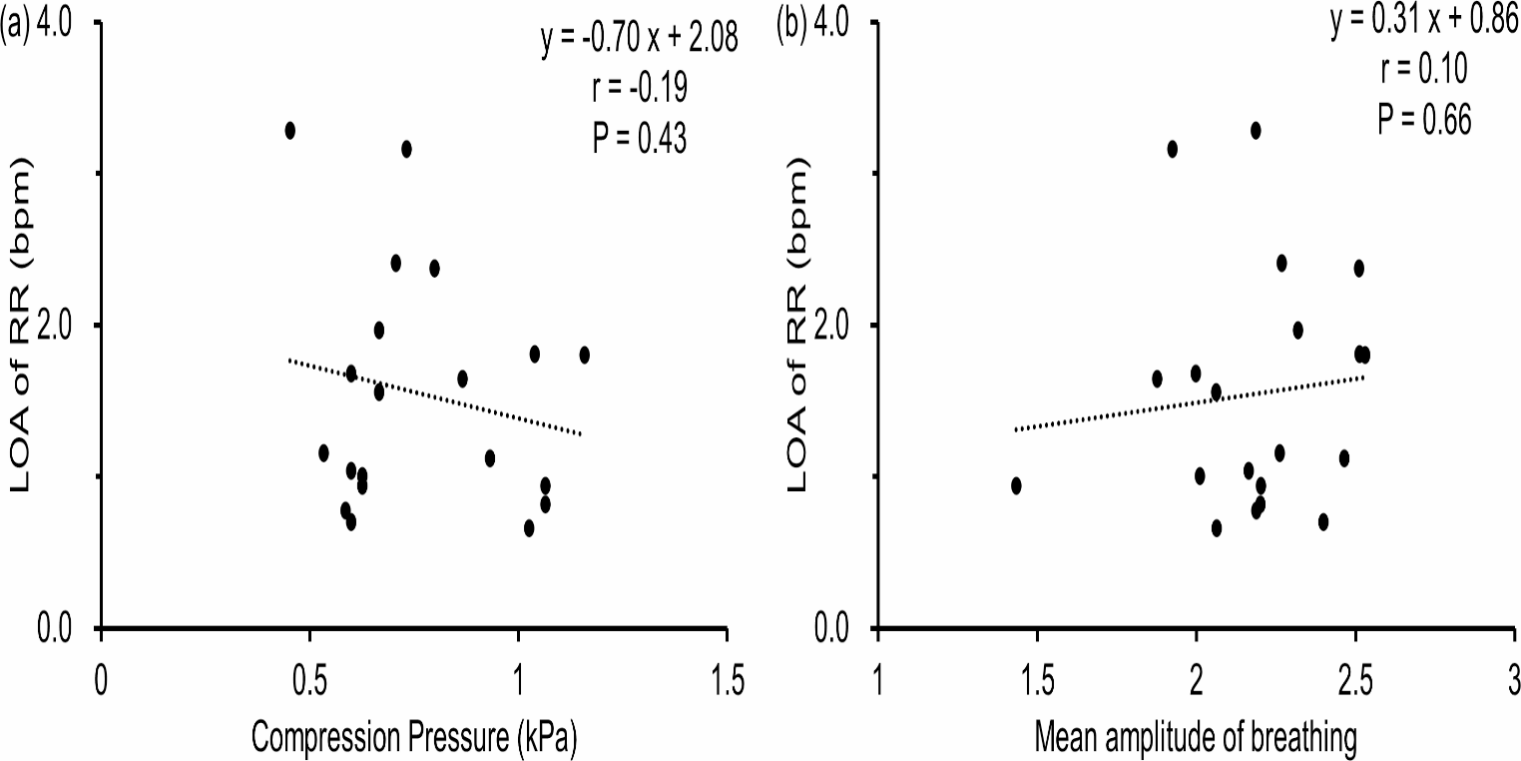
Limit of agreement (LOA) versus compression pressure (**a**) and mean amplitude of breathing (**b**). Each scatter point in both figures is the mean value for the sitting, supine, and lateral positions

In our proposed method for measuring respiratory rate using a capacitive sensor under low compression pressures, Fig. 8a presents no significant relationship between compression pressure and measurement error. The data indicate that even pressures ranging as 0.45–1.16 kPa are sufficient for accurate measurements. This is attributed to the principle of capacitance sensors utilized in previous studies, where the pressure applied due to changes in the thorax shape during respiration alters the distance between electrodes [36]. Another method involves positioning electrodes on the chest and back, capturing the change in distance and dielectric constant due to respiration [25]. For both methods, ensuring that the sensor and electrodes are tightly attached to the body with no gaps is typically crucial for accurate respiration measurement. Conversely, our proposed smart clothing integrates the sensor within the fabric, measuring the bending angle caused by respiration, thus allowing for accurate measurements even if gaps exist between the body and the sensor. Additionally, although low wearing pressure may cause the smart clothing to wrinkle due to changes in posture, the dual-layer structure of the sensor compensates for these wrinkles, ensuring they do not impact measurement accuracy. These findings affirm that the sensor is highly sensitive and capable of detecting changes in body shape even under low compression pressures, highlighting its effectiveness for respiratory rate measurement without stringent requirements on sensor-body contact.

The respiratory detection performance in the lateral position was evaluated only in the direction where the bending angle sensor was sandwiched between the bed and body. This condition was more severe than that in the opposite direction because the sensor was pressed; however, the respiratory rate could be measured at 0.0 ± 1.4 bpm. This is thought to be because the bending angle sensor used in this study had a five-layer structure that was almost unaffected by pressure [32] and because the shape of the rib cage and abdomen changed more when breathing occurred in the chest and abdomen than when breathing occurred on the back [35]; even when the bending angle sensor is fixed, accurately measuring breathing is thought to be feasible.

Six out of 55 data points presented an error (LOA) of 4 bpm or more. We identified two primary issues upon investigating the causes of measurement errors exceeding 4 bpm, which accounted for approximately 2% of the cases. The first issue was overcounting, where a single breath was mistakenly registered as two or more breaths. The second issue was undercounting, where multiple breaths were present, but only one was detected. In this study, we employed the zero-crossing method to determine respiration rates. However, baseline fluctuations in sensor output owing to artifacts could result in both overcounts and undercounts. We mitigated the influence of artifacts by processing signals using a combination of rib cage and abdominal sensors. Adopting a respiration detection algorithm more tolerant to baseline fluctuations could further decrease these errors.

This study had a few limitations. The study only included young healthy males; the results of this study could vary because of gender- and age-related differences in ventilation volume, and the associated differences in body shape changes during breathing [38]. However, because the proposed system does not need to be tightly fit and measures the respiratory rate at low compression, the measurement accuracy is not significantly affected by differences in body shape. The system was able to correctly measure the respiratory rate for a wide range of body shapes (Table 1). Furthermore, although the sensor length must be adjusted according to the rib cage and abdomen circumference, as shown in Eq. (7), sensor length is not related to sensitivity of the sensor output and does not affect the accuracy of the measurement. The change in antero-posterior diameters during respiration in females is approximately half that in males [33]. In this study, breathing could be measured for amplitudes as low as 0.3, compared with the mean amplitude of 2.2 for the ensemble waveform. Moreover, no significant correlation was observed between the amplitude and the LOA of the measurement error (Fig. 8b). Therefore, the system can potentially measure accurately even if the ventilation volume is decreased by half. Before clinical application of the proposed system, we plan to verify whether the system can accurately measure the respiratory rate in patients with chronic respiratory diseases regardless of their gender and age. These verifications will enable self-monitoring of respiratory rate in patients with chronic respiratory disease, and clinically useful indicators can measure respiratory rate without the use of excessive pressure. Continuous self-monitoring of vital signs at home, which requires only the wearing of a smart wear, could help in the early detection of symptom exacerbations and contribute to improved compliance with treatment of disease symptoms.

## Conclusions

This study proposed a method for measuring respiratory rate using smart clothing equipped with a bending angle sensor based on double-layer capacitance. This method accurately measured resting respiration rates ranging within 12–30 bpm with practically acceptable accuracy. The smart clothing facilitated respiratory rate measurement without causing discomfort to the user and was minimally influenced by the user’s body shape or measurement posture. Consequently, this smart clothing, utilizing the bending angle sensor based on double-layer capacitance, demonstrate considerable potential as a method for the quantitative evaluation of respiratory rate during rest. In addition, smart clothing that utilizes a bending angle sensor based on double-layer capacitance can measure body shape changes with breathing in the rib cage and abdomen, and respiratory rate can be measured without any effects of uncomfortable compression. However, the proposed method exhibited measurement errors of 4 bpm or greater owing to artifacts such as body movement. In the future, the addition of a respiration detection algorithm that reduces the effects of artifacts may reduce the error and improve the stability of the measurement. The ability of the system to measure not only resting breaths but also coughs and irregular breaths will be examined to indicate its potential use for continuous self-monitoring of respiratory rate and disease symptoms in patients with chronic respiratory disease without uncomfortable pressure feeling. The proposed system is expected to aid in the early detection of symptom exacerbations and improve compliance with treatment of disease symptoms through continuous monitoring of vital signs.
